# The Efficacy of Hellebrigenin Against Nasopharyngeal Carcinoma Cells: The Molecular and Bioinformatic Analysis

**DOI:** 10.1111/jcmm.70624

**Published:** 2025-05-28

**Authors:** Hsin‐Yu Ho, Mu‐Kuan Chen, Yun‐Jung Tsai, Chia‐Chieh Lin, Yu‐Sheng Lo, Yi‐Ching Chuang, Ming‐Ju Hsieh

**Affiliations:** ^1^ Oral Cancer Research Center Changhua Christian Hospital Changhua Taiwan; ^2^ Department of Otorhinolaryngology, Head and Neck Surgery Changhua Christian Hospital Changhua Taiwan; ^3^ Graduate Institute of Clinical Medicine, College of Medicine National Chung Hsing University Taichung Taiwan; ^4^ Department of Surgical Pathology Changhua Christian Hospital Changhua Taiwan; ^5^ Translational Pathology Core Laboratory Changhua Christian Hospital Changhua Taiwan; ^6^ Graduate Institute of Biomedical Sciences China Medical University Taichung Taiwan

**Keywords:** apoptosis, CHCHD2, hellebrigenin, MAPKs pathway, nasopharyngeal carcinoma

## Abstract

Nasopharyngeal carcinoma (NPC) is a unique cancer type originating from the nasopharynx. To investigate novel strategies for improving prognosis and reducing the adverse effects of current treatments, this study examined the efficacy of hellebrigenin. Hellebrigenin demonstrated selective cytotoxicity against NPC‐BM and NPC‐039 cell lines without harming normal nasopharyngeal cells. Treatment with hellebrigenin resulted in G2/M cell cycle arrest in both NPC cell lines. The apoptotic phenomena induced by hellebrigenin included chromatin condensation, increased apoptotic cells and altered mitochondrial membrane potential. Proteomics analysis and the bioinformatic data identified coiled‐coil‐helix‐coiled‐coil‐helix domain containing 2 (CHCHD2) as a candidate oncogene in NPC. Moreover, the combination of CHCHD2 siRNA, CHCHD2 plasmid and hellebrigenin pointed out that CHCHD2 could be a critical mediator of hellebrigenin‐induced apoptosis. The combined treatment of hellebrigenin with mitogen‐activated protein kinase inhibitors revealed the involvement of the extracellular signal–regulated kinases and c‐Jun N‐terminal kinases pathways in hellebrigenin‐induced apoptosis in NPC cells. In vivo studies demonstrated that hellebrigenin suppressed the tumour volume without affecting body weight, accompanied by the downregulation of Ki67 and CHCHD2 expression. In conclusion, this study provides evidence that hellebrigenin induces NPC apoptosis through regulating CHCHD2 both in vitro and in vivo.

**Abbreviations**
CDC2: cell division cycle protein 2 homologue
CDK: cyclin‐dependent kinase
CHCHD2: coiled‐coil‐helix‐coiled‐coil‐helix domain containing 2
DAPI: 4′,6‐Diamidino‐2‐phenylindole
DEGs: differentially expressed genes
DR5: death receptor 5
ERK: extracellular signal–regulated kinases
FAS: Fas cell surface death receptor
FITC: fluorescein isothiocyanate
GO: Gene Ontology
HNSCC: head and neck squamous cell carcinoma
iTRAQ: isobaric tags for relative and absolute quantitation
JNK: c‐Jun N‐terminal kinases
KEGG: Kyoto Encyclopedia of Genes and Genomes
MAPKs: mitogen‐activated protein kinases
MTT: 3‐(4, 5‐Dimethylthiazol‐2‐yl)‐2, 5‐diphenyltetrazolium bromide
NPC: nasopharyngeal carcinoma
PARP: poly (ADP‐ribose) polymerase
PI: propidium iodide
RIP: receptor‐interacting protein
TCGA: The Cancer Genome Atlas
TME: tumour microenvironment

## 1 Introduction

Nasopharyngeal carcinoma (NPC) is a distinct cancer type primarily originating from the nasopharyngeal epithelium. Although NPC exhibits a global incidence rate of < 1 per 100,000 person‐years, it is notably prevalent in specific regions, such as East and Southeast Asia as well as parts of North Africa [1]. In Taiwan, however, the incidence rate of NPC has been reported to be as high as 1.94–6.75 per 100,000 person‐years in 2021 according to national cancer registry data [2]. The standard treatment for patients with stage I NPC is radiotherapy, whereas concurrent chemoradiation is recommended for those with advanced stages [3, 4]. Although the combination of radiotherapy and chemotherapy (cisplatin or fluorouracil) can improve prognosis and enhance survival rates in patients with NPC [4], these treatments often have considerable side effects and limitations, particularly in cases of recurrence and metastasis. Consequently, the identification of innovative therapeutic approaches that offer higher efficacy and lower toxicity is paramount.

Bufadienolides, a class of steroidal compounds, are widely distributed in toad venom and certain plant species, including the Ranunculaceae *Helleborus* and Asparagaceae *Scilla* and *Bowiea* in the Scilloideae subfamily [5]. These compounds exhibit potent cardiotonic effects due to their ability to inhibit Na^+^/K^+^‐ATPase, an enzyme essential for maintaining ion balance in cardiac cells. Bufadienolides have also been demonstrated to exert anticancer effects through various mechanisms, including the suppression of epithelial–mesenchymal transition [6], induction of apoptosis [7] and disruption of the cell cycle [8]. Among bufadienolides, hellebrigenin has been noted for its potent anticancer effects across various cancer types. Han et al. highlighted that hellebrigenin inhibited cancer cell survival by downregulating the levels of cell cycle proteins, thus exerting cytotoxicity in cancerous glial cells [9]. In oestrogen receptor‐positive and triple‐negative breast cancers, hellebrigenin exerted cytotoxicity through G2/M phase arrest and the modulation of mitochondrial function and its influence on apoptotic pathways [10]. Additionally, Wei et al. reported that hellebrigenin can inhibit cell proliferation in pancreatic carcinoma by activating autophagy and apoptosis [11]. However, the specific effects of hellebrigenin in NPC cells remain understudied. This study investigated the apoptotic effects of hellebrigenin on NPC cells, focusing on its interaction with coiled‐coil‐helix‐coiled‐coil‐helix domain containing 2 (CHCHD2). By elucidating the underlying mechanism of action, this study seeks to establish a foundational understanding for the development of therapies in the future. The study findings may contribute to the development of novel therapeutic strategies targeting mitochondrial pathways for cancer treatment.

## 2 Materials and Methods

### 2.1 Chemicals

**FIGURE 1:**
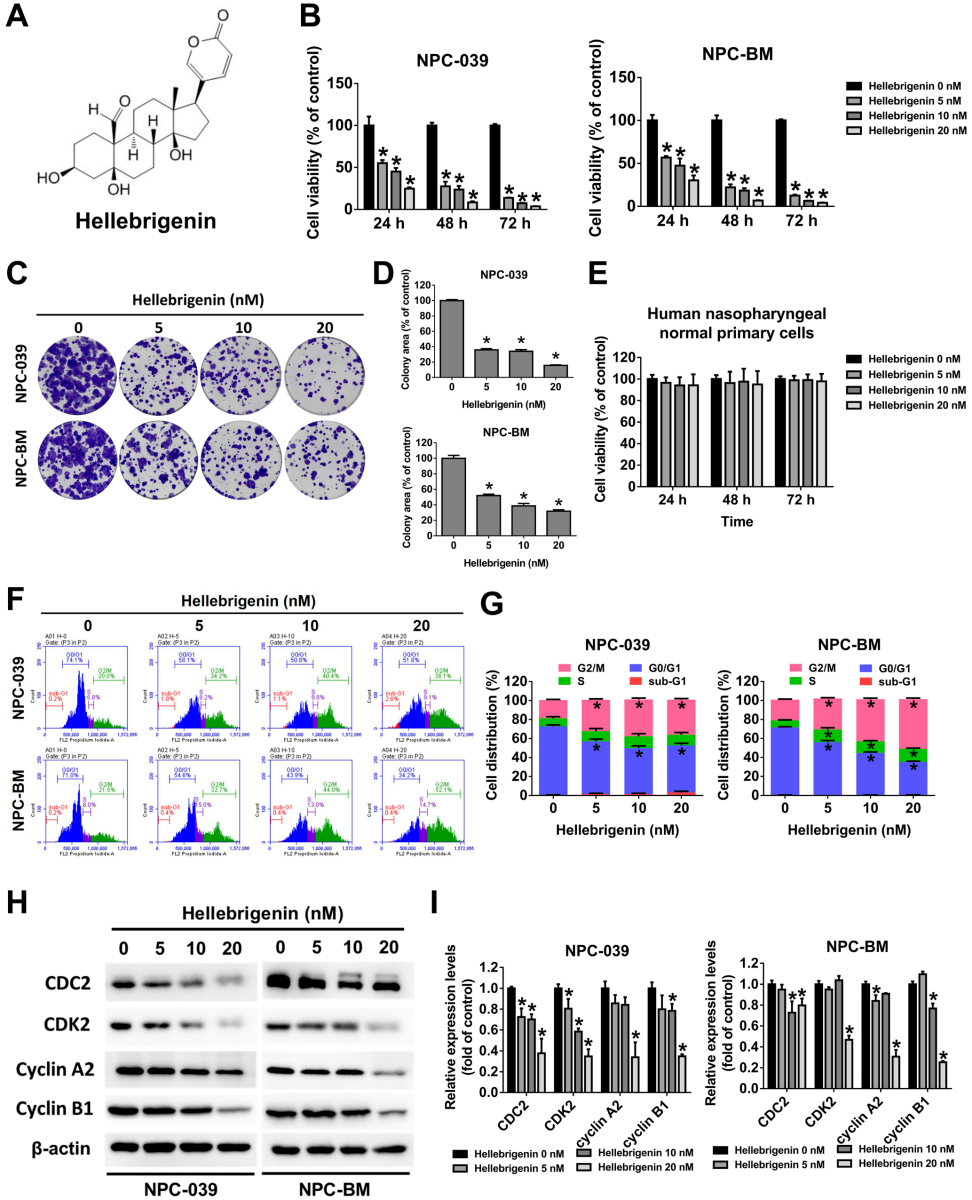
Cytotoxic effects of hellebrigenin on NPC cell lines. (A) Chemical structure of hellebrigenin. (B) Viability of NPC‐039 and NPC‐BM cells was measured using the MTT assay following treatment of the cells with hellebrigenin (0, 5, 10 and 20 nM) for 24, 48 or 72 h. The results are presented as mean ± SD, *n* = 3. *p < 0.05 compared with 0 nM. (C, D) Colony formation in NPC cell lines treated with hellebrigenin was assessed using a colony formation assay. The results are presented as mean ± SD, *n* = 3. *p < 0.05 compared with 0 nM. (E) Viability of human normal nasopharyngeal primary cells was measured using the MTT assay following treatment with hellebrigenin (0, 5, 10 and 20 nM) for 24 h. The results are presented as mean ± SD, *n* = 3. *p < 0.05 compared with 0 nM. (F, G) Cells treated with 0, 5, 10 and 20 nM of hellebrigenin for 24 h were collected and analysed through flow cytometry. The results are presented as mean ± SD, *n* = 3. *p < 0.05 compared with 0 nM. (H, I) Cell cycle proteins CDC2, CDK2, cyclin A2 and cyclin B1 were measured using a Western blot assay. The results are presented as mean ± SD, *n* = 3. *p < 0.05 compared with 0 nM.

Hellebrigenin (purity ≥ 99%, Figure 1A) and mitogen‐activated protein kinase (MAPK) inhibitors (U0126 and JNK‐in‐8) were purchased from MedChemExpress (Monmouth Junction, NJ, USA). All stock solutions were prepared with dimethyl sulfoxide (DMSO). The stock solutions prepared with DMSO at a final concentration of < 0.2% were used in all experiments.

### 2.2 Cell Lines

The human NPC cell lines NPC‐BM and NPC‐039 are, respectively, derived from Taiwanese [12] and Chinese patients [13]. These cell lines were obtained from Dr. Jen‐Tsun Lin (Haematology and Oncology, Changhua Christian Hospital). The cells were cultured in Roswell Park Memorial Institute‐1640 medium (RPMI‐1640; Gibco BRL, Grand Island, NY, USA) supplemented with penicillin, streptomycin, sodium pyruvate and 10% foetal bovine serum (Merck Millipore, Burlington, MA, USA). A normal human nasopharyngeal primary cell line purchased from Celprogen Inc. (Cat# 36073‐04; Torrance, CA, USA) was incubated under the conditions recommended by the manufacturer.

### 2.3 Assessment of Cell Viability

After hellebrigenin treatment (24, 48 or 72 h) or transient transfection, cells were incubated for 2 h with the working solution of 3‐(4, 5‐dimethylthiazol‐2‐yl)‐2, 5‐diphenyltetrazolium bromide (MTT; Sigma‐Aldrich, St. Louis, MO, USA). Subsequently, the formed formazan crystals were dissolved in methanol and analysed on a microplate reader (BioTek, Winooski, VT, USA). The OD was measured at 570 nm.

### 2.4 Colony Formation Assay

For colony formation, 5 × 10^2^ cells in a six‐well plate were incubated with hellebrigenin (0, 5, 10 or 20 nM) for 24 h. Subsequently, the medium was replaced with fresh medium every 3 days until 14 days. The growth colonies were fixed and stained separately with methanol and Giemsa staining solution (Sigma‐Aldrich). Subsequently, the area of stained colonies was analysed through ImageJ (National Institutes of Health, USA).

### 2.5 Flow Cytometry Analysis

For the cell cycle assay, after hellebrigenin treatment for at least 3 h, cells were collected and fixed with 70% ethanol. The ethanol was discarded, and the cells were then incubated with propidium iodide (PI) solution (BD Biosciences, Franklin Lakes, NJ, USA) at room temperature for 30 min. For the apoptosis assay, the cells were collected and stained with fluorescein isothiocyanate (FITC) annexin V and PI solution (BD Biosciences) at room temperature. For the mitochondrial membrane potential assay, the cells were collected and stained with MitoScreen JC‐1 solution (BD Biosciences) at 37°C. The samples from all experiments were detected by BD Accuri C6 Plus flow cytometer (BD Biosciences), and the data were analysed using BD CSampler Plus software (version 1.0; BD Biosciences).

### 2.6 Western Blot Assay

Proteins were extracted from cells treated with hellebrigenin (0, 5, 10 or 20 nM) for 24 h. An equal amount of proteins (20 μg) from each group was separated through 12% SDS‐PAGE for 2 h. The separated proteins were then transferred to a 0.22‐μm polyvinylidene difluoride membrane (Merck Millipore). After being blocked with 5% skim milk at room temperature for 1 h, the membranes were washed and incubated with the indicated primary antibody overnight. Primary antibodies against cell division cycle protein 2 homologue (CDC2, [#9116]), cyclin‐dependent kinase 2 (CDK2, [#2546]), cyclin A2 (#4656), cyclin B1 (#12231), β‐actin (#4970), death receptor 5 (DR5, [#8074]), Fas cell surface death receptor (FAS, [#4233]), receptor‐interacting protein (RIP; #3493), Bcl‐xL (#2764), Mcl‐1 (#94296), cleaved (c)‐caspase 3 (#9664), c‐caspase 8 (#9496), c‐caspase 9 (#52873), c‐poly (ADP‐ribose) polymerase (c‐PARP; #5625), extracellular signal–regulated kinases (ERK, [#4695]) phosphorylated (p)‐ERK (#4370), p38 (#9212), c‐jun N‐terminal kinases (JNK, [#9258]) and p‐JNK (#4668) were purchased from Cell Signalling Technology (Danvers, MA, USA). Primary antibodies against Bak (#06‐536) and p‐p38 (#44‐684G) were purchased from Merck Millipore and Invitrogen (Carlsbad, MA, USA), respectively. A primary antibody against CHCHD2 (#19424‐1‐AP) was purchased from Proteintech (Rosemont, IL, USA). The dilution factor for all primary antibodies was 1:1000. The membranes were then incubated with either anti‐mouse or anti‐rabbit secondary antibody (1:10,000 dilution; Jackson ImmunoResearch, West Grove, PA, USA). Blots were visualised with a WesternBright Sirius HRP substrate (Advansta Inc., San Jose, CA, USA) and measured using the ChemiDoc MP Imaging System (Bio‐Rad, Hercules, CA, USA).

### 2.7 Nuclear Staining Assay

After 24‐h treatment with hellebrigenin, cells in a six‐well dish were fixed with methanol and stained with a blue‐fluorescent DNA stain (4′,6‐diamidino‐2‐phenylindole [DAPI]). Fluorescence was recorded using a fluorescence microscope (Leica Biosystems Division of Leica Microsystems Inc., Buffalo Grove, IL, USA).

### 2.8 Proteomics Analysis

An equal amount of proteins from each group of cells, treated or not treated with hellebrigenin (10 nM) for 24 h, was diluted in 200 mM triethylammonium bicarbonate and reduced. The samples were then digested with sequencing‐grade modified porcine trypsin (Promega, Madison, WI, USA) at 37°C for 16 h. The resulting peptides were labelled with isobaric tags for relative and absolute quantitation (iTRAQ), pooled and desalted. The peptides were analysed with the LC apparatus coupled to a 2D linear ion trap mass spectrometer (LTQ‐Orbitrap ELITE; Thermo Fisher Scientific) operated using Xcalibur 2.2 software (Thermo Fisher Scientific). Data analysis was conducted using Proteome Discoverer software (version 2.3, Thermo Fisher Scientific), and the reporter ions quantifier node was used for iTRAQ quantification. The MS/MS spectra were searched against the SwissProt database. Differential proteins were identified using a threshold based on the mean of the log2‐transformed ratio ± 1 standard deviation of the log2‐transformed ratio, assuming a normal distribution of the normalised ratio.

### 2.9 Transient Transfection Assay

Human siRNAs (scrambled control and *CHCHD2*) were purchased from Cohesion Biosciences (London, UK). The pCMV3‐N‐GFPSpark vector for negative control and the human CHCHD2 clone vector were purchased from Sino Biological Inc. (Beijing, China). The transient transfection assay was performed using TransIT‐X2 reagent (Mirus Bio; Madison, WI, USA) in accordance with the manufacturer's instructions. For analysis, NPC cells were transfected with 2 μg of plasmid DNA or 25 nM siRNA for 24 h, with or without hellebrigenin treatment. Following treatment, the cells were collected for the assessment of cytotoxicity, protein/RNA expression or apoptosis.

### 2.10 Bioinformatic Analysis

The CHCHD2 expression data and clinical information of head and neck squamous cell carcinoma (HNSCC) patients were downloaded from the Cancer Genome Atlas (TCGA) database through the University of California Santa Cruz platform Xena (accessed July 2024) [14]. Only primary tumour and adjacent normal tissue samples with complete CHCHD2 expression, pathological stage, tumour size and survival data were included in the analysis. RNA‐seq data were normalised and log2‐transformed using the formula log2 (norm_count + 1) prior to downstream analyses. Additionally, the gene expression data set for NPC was retrieved from the GSE53819 data set (platform GPL6480) in the Gene Expression Omnibus (GEO) database. Both primary tumour tissues and non‐cancerous nasopharyngeal epithelial samples were included. As all data were obtained from publicly available databases, no institutional ethical approval was required.

### 2.11 Tumour Xenograft Assay

A human NPC xenograft mouse model was established by transplanting NPC‐BM cells (2 × 10^6^ cells/200 μL PBS + matrigel) into the right flank of 6‐week‐old NOD/SCID mice (BioLASCO Taiwan Co. Ltd.). Six mice were randomly divided into two groups (three mice in each group). Tumour cell injection was conducted for 7 days, and the eighth day was denoted the first experimental day (Day 0). Mice were intraperitoneally administered vehicle control or 4 mg/mL hellebrigenin twice weekly for 5 weeks. The body weight of the mice and the tumour size were measured twice weekly. Tumour size was measured by calliper, and the tumour volume was calculated using the ellipsoid equation: (length × width × width)/2. The mice were sacrificed at the end of the experiment (Day 35). Subsequently, the tumours were resected and fixed in neutral buffered formalin at room temperature. The experiment was approved by the Institutional Animal Care and Use Committee (IACUC‐2023‐SH‐018). Immunohistochemical (IHC) analysis was conducted on paraffin‐embedded tissue samples. The samples were sectioned, followed by deparaffinisation, antigen retrieval and blocking procedures prior to IHC staining. The primary antibodies against Ki67 (#ab15580; 1:2000 dilution) and CHCHD2 (#19424‐1‐AP; 1:200 dilution) were purchased from Abcam (Cambridge, UK) and Proteintech, respectively. After incubation with the primary antibody, the samples were incubated with an anti‐rabbit secondary antibody and subsequently imaged using a microscope (Leica Biosystems Division of Leica Microsystems Inc.).

### 2.12 Statistical Analysis

Statistical analyses were performed using Student's *t* test or one‐way ANOVA with GraphPad Prism V6.0 (GraphPad Software Inc.). A *p*‐value of < 0.05 was considered significant.

## 3 Results

### 3.1 Cytotoxic Effects of Hellebrigenin on NPC‐039 and NPC‐BM Cell Lines

To evaluate the cytotoxic effects of hellebrigenin on NPC cells, we treated the NPC‐039 and NPC‐BM cell lines with varying concentrations (0, 5, 10 and 20 nM) of hellebrigenin for different incubation periods (24, 48 and 72 h). As depicted in Figure 1B, hellebrigenin significantly reduced cell viability in a time‐ and dose‐dependent manner. Moreover, colony formation assays revealed that hellebrigenin suppressed the tumour‐forming ability of both NPC‐039 and NPC‐BM cell lines (Figure 1C,D). To assess the drug's selectivity, we examined the cytotoxic effects of hellebrigenin on a normal nasopharyngeal primary cell line. The results indicated that hellebrigenin exhibited selective cytotoxicity toward NPC cells but did not affect the viability of normal nasopharyngeal primary cells (Figure 1E). Flow cytometry analysis was employed to investigate the impact of hellebrigenin on cell cycle progression. Hellebrigenin treatment led to a decrease in the cell distribution in the G0/G1 phase and an accumulation of cells in the G2/M phase in NPC‐039 and NPC‐BM cell lines (Figure 1F,G). Notably, the accumulation of cells in the S phase was only observed in NPC‐BM cells. A Western blot analysis further confirmed the downregulation of the levels of cellcycle‐related proteins, including CDC2 (also known as CDK1), CDK2, cyclin A2 and cyclin B1, following exposure to different doses of hellebrigenin (Figure 1H,I).

### 3.2 Apoptosis Induction for NPC Cells by Hellebrigenin

**FIGURE 2:**
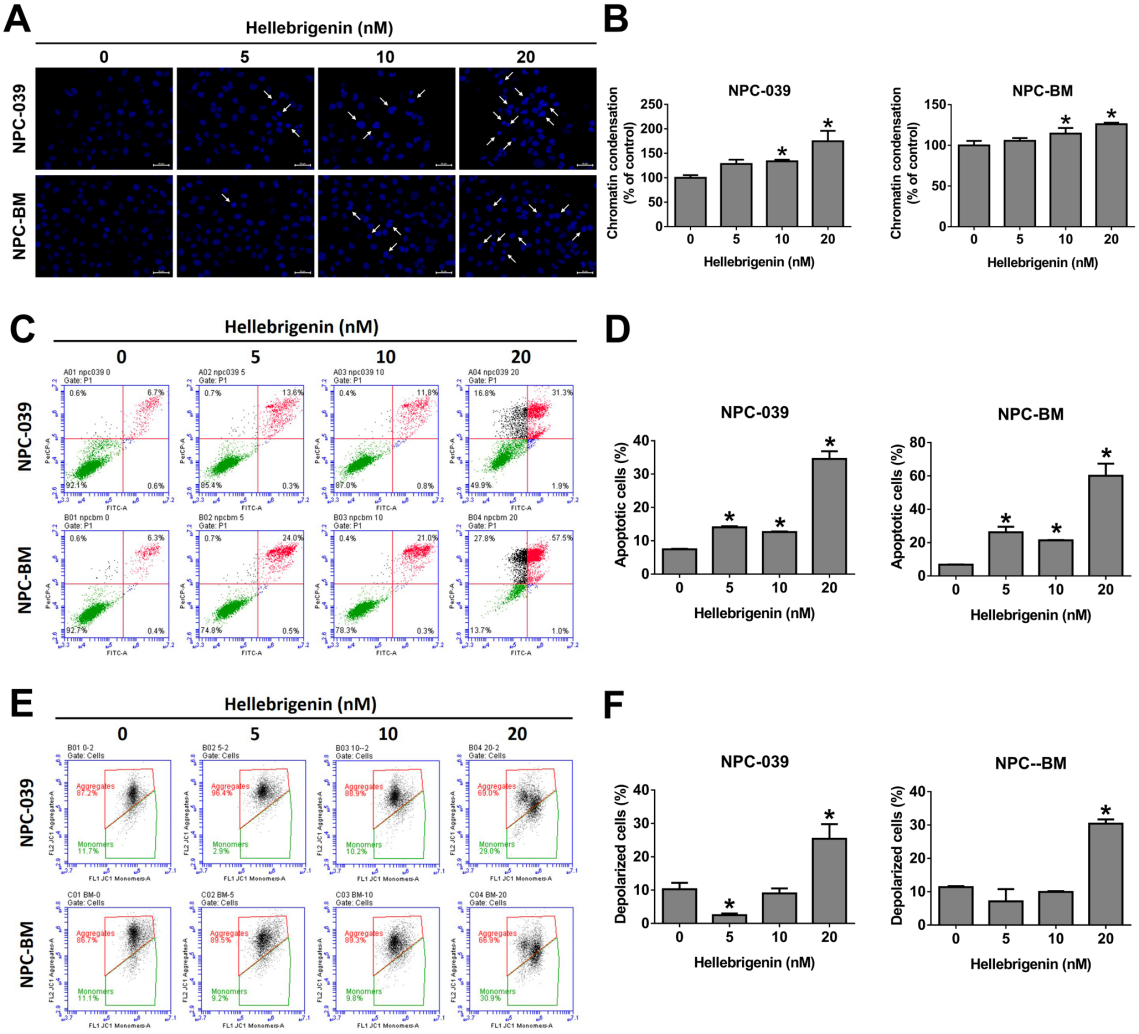
Apoptotic effects of hellebrigenin on NPC cell lines. (A, B) Chromatin condensation was assessed using DAPI staining following treatment of cells with hellebrigenin (0, 5, 10 and 20 nM) for 24 h. Scale bar = 50 μm. White arrows denote chromatin condensation. The results are presented as mean ± SD, *n* = 3. *p < 0.05 compared with 0 nM. (C, D) Cells treated with 0, 5, 10 or 20 nM of hellebrigenin were analysed using annexin V and PI staining through flow cytometry. The FITC (+) cells are identified as apoptotic cells. The results are presented as mean ± SD, *n* = 3. *p < 0.05 compared with 0 nM. (E, F) Cells treated with 0, 5, 10 or 20 nM of hellebrigenin were analysed using JC‐1 staining through flow cytometry. The monomers are identified as depolarized cells. The results are presented as mean ± SD, *n* = 3. *p < 0.05 compared with 0 nM.

**FIGURE 3:**
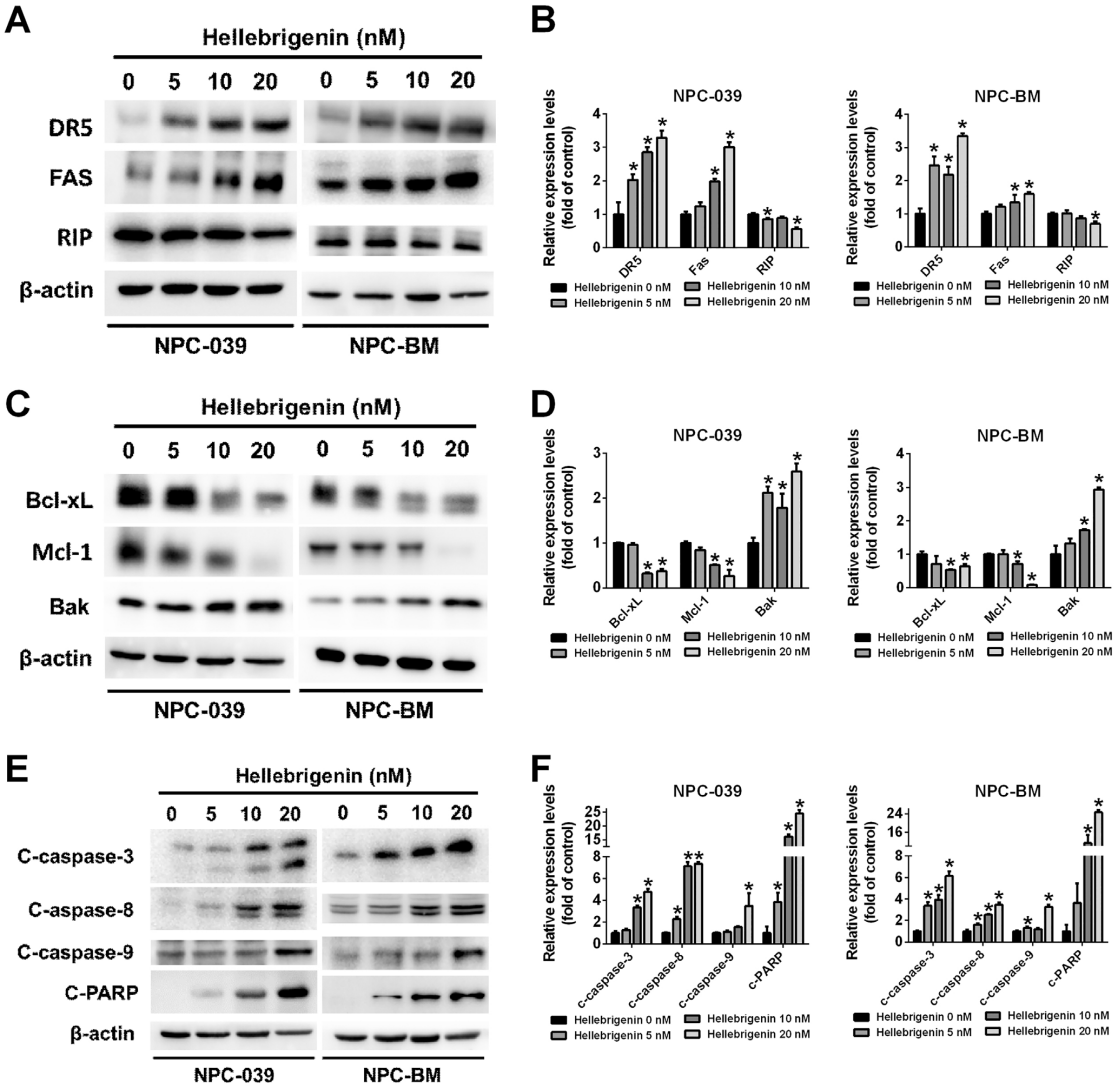
Effects of hellebrigenin on the intrinsic and extrinsic apoptotic pathways in NPC cells. (A, B) Death receptor proteins (DR5, FAS and RIP), (C, D) mitochondrial‐related proteins (Bcl‐xL, Mcl‐1 and Bak), (E, F) caspases (c‐caspase‐3, ‐8 and ‐9) and c‐PARP were measured using a Western blot assay following hellebrigenin treatment (0, 5, 10 and 20 nM). β‐Actin was used as the internal control. The results are presented as mean ± SD, *n* = 3. *p < 0.05 compared with 0 nM.

To investigate whether hellebrigenin induces cytotoxicity through apoptosis, we examined chromatin condensation, mitochondrial membrane potential and the expression of apoptotic‐related proteins. As depicted in Figure 2A,B DAPI staining revealed increased chromatin condensation in hellebrigenin‐treated cells. Additionally, annexin V/PI staining demonstrated an increased distribution of both early and late apoptotic cells in hellebrigenin‐treated groups compared with the vehicle group (Figure 2C,D). Furthermore, the mitochondrial membrane potential of NPC cells increased with high‐dose hellebrigenin treatment (Figure 2E,F). Apoptosis is initiated by the binding of their ligands to the cell surface death receptor (extrinsic pathway) or by the triggering of changes in the mitochondrial outer membrane potential by proapoptotic proteins (intrinsic pathway), leading to the activation of caspases and subsequent cell death [15]. As depicted in Figure 3A,B hellebrigenin treatment resulted in the increased expression of the death receptor proteins DR5 and Fas while suppressing RIP expression. Regarding the intrinsic pathway, hellebrigenin downregulated the expression of Bcl‐xl and Mcl‐1 and upregulated the expression of Bak (Figure 3C,D). Moreover, the levels of cleaved caspase proteins were significantly elevated in the hellebrigenin‐treated groups (Figure 3E,F). This finding indicates that hellebrigenin may activate apoptosis through both the intrinsic and extrinsic pathways.

### 3.3 The Key Role of CHCHD2 in Hellebrigenin‐Treated NPC Cells

**FIGURE 4:**
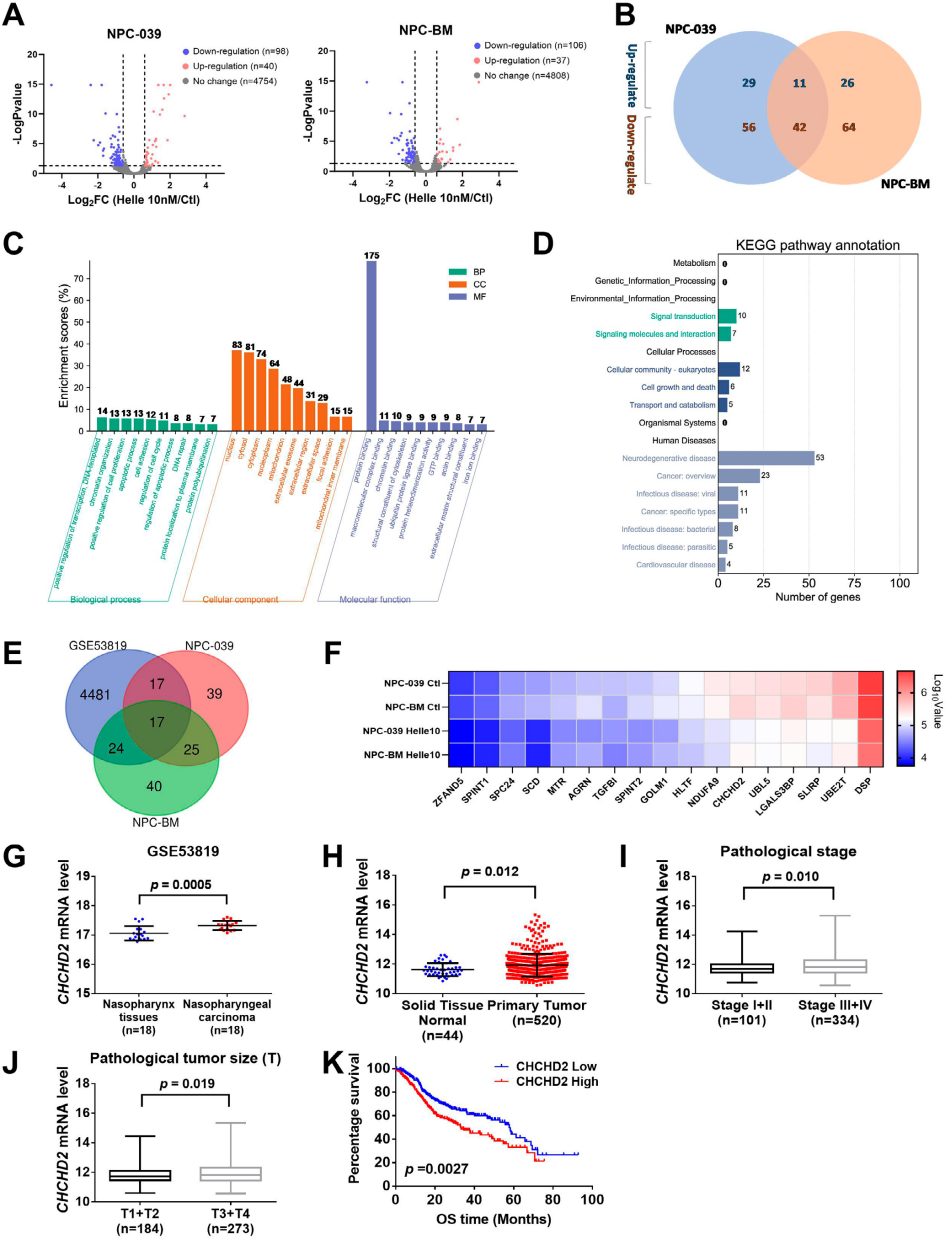
The proteomics analysis of NPC cell lines treated with hellebrigenin and bioinformatic analysis of clinical data. (A) Volcano plots illustrating DEGs identified using a cut‐off of adjusted *p* < 0.05 and log2 fold change ≥ 0.6 or ≤ −0.6. Ninety‐eight genes were identified to be downregulated, and 40 genes were identified to be upregulated in the NPC‐039 cell line. By contrast, in the NPC‐BM cell line, 106 genes were downregulated, and 37 genes were upregulated. (B) Venn diagram illustrating the overlap of DEGs between the two cell lines. (C) GO analysis of hellebrigenin‐regulated genes. Top 10 DEGs associated with biological processes, cellular components and molecular function were selected. Numbers above each bar denote the number of genes identified. (D) Enrichment analysis by using KEGG pathway annotations. DEGs associated with environmental information processing, cellular processing and human disease were selected. (E) Venn diagram illustrating the overlap of DEGs between the GSE53819 data set of NPC patients and downregulated genes of two NPC cell lines. (F) Heatmap presenting the 17 downregulated genes in the NPC‐039, NPC‐BM cell lines and GSE data set. (G) Comparison of CHCHD2 mRNA expression levels in normal tissues versus tumour tissues of NPC from the GSE53819 data set. (H) Comparison of CHCHD2 mRNA expression levels in normal tissues versus tumour tissues of HNSCC patients from the TCGA database. (I) Comparison of CHCHD2 mRNA expression levels in earlier versus advanced stages of HNSCC patients from the TCGA database. (J) Comparison of CHCHD2 mRNA expression levels in different tumour sizes of HNSCC patients from the TCGA database. (K) Comparison of CHCHD2 mRNA expression levels in patients with low versus high CHCHD2 expression in terms of overall survival from the TCGA database.

To identify differentially expressed genes (DEGs) influenced by hellebrigenin treatment, NPC‐039 and NPC‐BM cells were treated with either a vehicle control or 10 nM hellebrigenin and were subsequently analysed using the iTRAQ assay. The volcano plots reveal the DEGs in the hellebrigenin‐treated groups of NPC‐039 and NPC‐BM cells, with downregulated genes depicted in blue and upregulated genes in red (Figure 4A). A Venn diagram illustrates the overlap of 11 upregulated and 42 downregulated genes between NPC‐039 and NPC‐BM cells (Figure 4B). To elucidate the functions of these DEGs, enrichment analyses were performed using Gene Ontology (GO) and Kyoto Encyclopedia of Genes and Genomes (KEGG) pathway annotations. GO analysis indicated that the DEGs were enriched in cellular components, such as the nucleus, cytosol, cytoplasm, mitochondrion and extracellular exosomes, biological processes, such as apoptotic process and regulation of cell cycle as well as in the molecular function related to protein binding (Figure 4C). The KEGG pathway analysis revealed enrichment in signal transduction, cellular community, cell growth and death, neurodegenerative disease and cancer overview (Figure 4D).

To identify the genes affected by hellebrigenin in NPC, we integrated the GSE53819 data set with NPC‐039 and NPC‐BM data sets. Our analysis revealed that 17 downregulated genes in NPC‐039 and NPC‐BM overlapped with genes that exhibited higher expression levels in nasopharyngeal carcinoma patients within the GSE53819 data set (Figure 4E). As shown in Figure 4F, a heatmap was generated based on the 17 genes identified in NPC‐039 and NPC‐BM cells. CHCHD2 is a key regulator of mitochondrial homeostasis, which localised in the mitochondrial intermembrane space, and has been implicated in apoptosis activation [16, 17]. To elucidate the correlation between CHCHD2 and the pathological status of patients with NPC and HNSCC, data from TCGA for HNSCC and GSE53819 for NPC were analysed. The analysis demonstrated significantly higher CHCHD2 mRNA expression in primary tumours compared to normal tissues in both the GSE53819 NPC data set (Figure 4G) and the TCGA HNSCC data set (Figure 4H). Additionally, CHCHD2 mRNA expression was higher in advanced stages of HNSCC than in earlier stages (Figure 4I), with a similar trend observed for tumour size (Figure 4J). The survival curve demonstrated a strong correlation between high CHCHD2 expression and low survival (Figure 4K). Collectively, proteomic analysis suggests that hellebrigenin may induce apoptosis in NPC cells via the mitochondrial pathway. Among the regulated genes, CHCHD2 was found to be positively correlated with advanced clinical features of HNSCC patients and associated with poor prognosis. Therefore, we propose that CHCHD2 plays a critical role in hellebrigenin‐induced apoptosis in NPC cells.

### 3.4 Involvement of CHCHD2 in Hellebrigenin‐Induced Apoptosis

**FIGURE 5:**
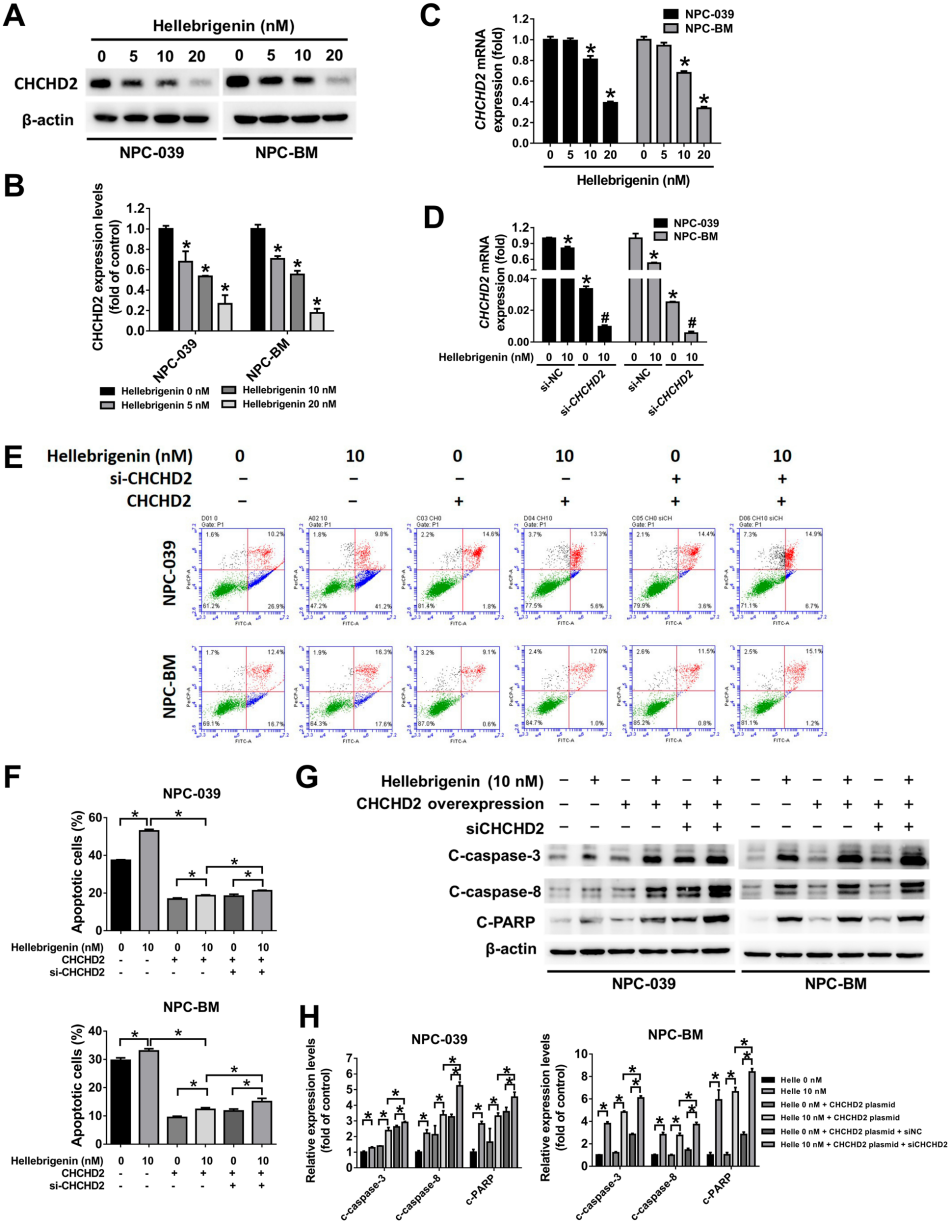
Involvement of CHCHD2 in hellebrigenin‐induced apoptosis in NPC cells. (A, B) CHCHD2 protein expression in hellebrigenin‐treated NPC cells. β‐Actin was identified as the internal control. The results are presented as mean ± SD, *n* = 3. *p < 0.05 compared with 0 nM. (C) mRNA levels of CHCHD2 in NPC cells treated with hellebrigenin. The results are presented as mean ± SD, *n* = 3. *p < 0.05 compared with 0 nM. (D) mRNA levels of CHCHD2 in NPC cells treated with hellebrigenin with or without CHCHD2 siRNA. The results are presented as mean ± SD, *n* = 3. *p < 0.05 compared with 0 nM. #*p* < 0.05 compared with hellebrigenin 10 nM. (E, F) Cells treated with hellebrigenin, CHCHD2 plasmid or CHCHD2 siRNA were analysed using annexin V and PI staining through flow cytometry. The FITC (+) cells are identified as apoptotic cells. The results are presented as mean ± SD, *n* = 3. *p < 0.05. (G, H) Protein levels of c‐caspase‐3, ‐8 and ‐9, and PARP were assessed in cells treated with hellebrigenin, CHCHD2 plasmid or CHCHD2 siRNA through a Western blot assay. β‐Actin was identified as the internal control. The results are presented as mean ± SD, *n* = 3. *p < 0.05.

To investigate the involvement of CHCHD2 in hellebrigenin‐induced apoptosis, we examined the effects of hellebrigenin on CHCHD2 expression at both the translational and transcriptional levels. The results revealed that hellebrigenin significantly suppressed the protein (Figure 5A,B) and mRNA (Figure 5C) expression levels of CHCHD2 in a dose‐dependent manner. To evaluate the role of hellebrigenin‐suppressed CHCHD2 expression in NPC cells, we implemented CHCHD2 overexpression and CHCHD2 siRNA knockdown experiments. The combination of hellebrigenin and CHCHD2 siRNA resulted in the more pronounced downregulation of CHCHD2 mRNA expression compared with hellebrigenin alone (Figure 5D). Annexin V/PI staining revealed that CHCHD2 overexpression rescued cells from apoptosis compared with the vehicle strategy. Conversely, the combination of CHCHD2 overexpression, CHCHD2 siRNA and hellebrigenin significantly increased the percentage of apoptotic cells (Figure 5E,F). Moreover, the expression levels of cleaved caspase‐3, caspase‐8 and PARP exhibited a similar trend to the apoptotic assay results (Figure 5G,H). This result suggests that CHCHD2 is involved in hellebrigenin‐induced apoptosis in NPC cells.

### 3.5 Suppression of ERK and JNK Pathways in Hellebrigenin‐Induced Apoptosis

**FIGURE 6:**
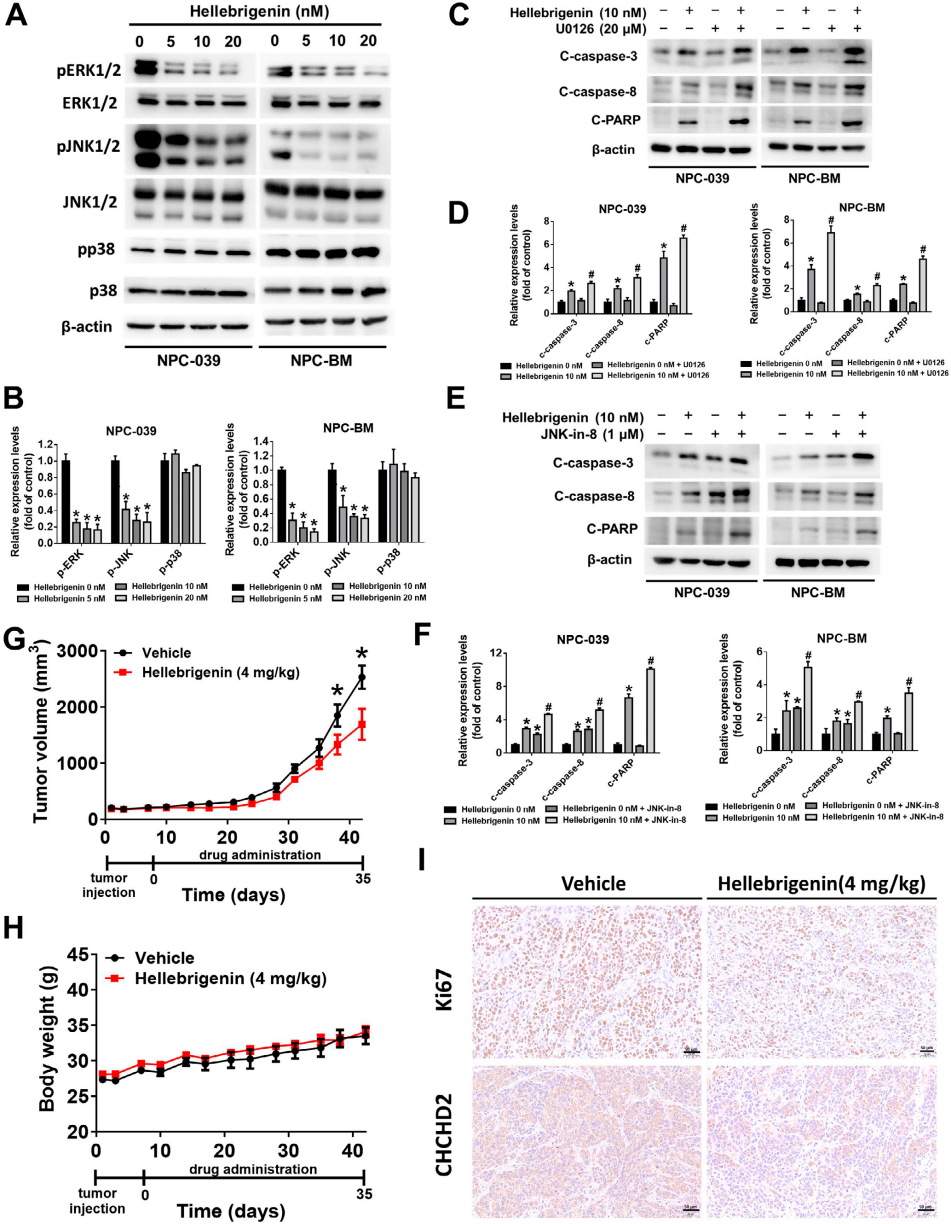
Effects of hellebrigenin on apoptosis in NPC cells and in an NPC xenograft model. (A, B) MAPK protein expression was assessed using a Western blot assay following hellebrigenin treatment (0, 5, 10 and 20 nM). β‐Actin was identified as the internal control. The results are presented as mean ± SD, *n* = 3. *p < 0.05 compared with 0 nM. (C, D) Cells were treated with 10 nM hellebrigenin with or without the ERK inhibitor U0126. β‐Actin was identified as the internal control. The results are presented as mean ± SD, *n* = 3. *p < 0.05 compared with 0 nM. #*p* < 0.05 compared with hellebrigenin 10 nM. (E, F) Cells were treated with 10 nM of hellebrigenin with or without the JNK inhibitor JNK‐in‐8. β‐Actin was identified as the internal control. The results are presented as mean ± SD, *n* = 3. *p < 0.05 compared with 0 nM. # *p* < 0.05 compared with hellebrigenin 10 nM. (G) Tumour volumes of the vehicle group and NPC xenograft mice treated with 4 mg/kg of hellebrigenin. The seventh day following tumour injection was identified as Day 0. The results are presented as mean ± SE, *n* = 3. *p < 0.05 compared with vehicle. (H) Body weights of the vehicle group and NPC xenograft mice treated with 4 mg/kg of hellebrigenin. The results are presented as mean ± SE, *n* = 3. *p < 0.05 compared with vehicle. (I) Immunohistochemical analysis of Ki67 and CHCHD2 expression in the vehicle group and NPC xenograft mice treated with 4 mg/kg of hellebrigenin. Scale bar = 50 μm.

MAPKs are serine‐/threonine‐specific protein kinases that transduce signals from the outer cellular membrane to the cytosol or nucleus, and these kinases play crucial roles in cellular functions such as inflammation, apoptosis, autophagy and cell division [18]. To investigate the involvement of MAPKs in hellebrigenin‐induced apoptosis, we examined the expression of ERK, JNK and p38 following 24‐h hellebrigenin treatment. As indicated in Figure 6A,B, the expression levels of p‐ERK and p‐JNK were significantly downregulated in NPC‐039 and NPC‐BM cells, whereas p‐p38 expression remained unchanged. To determine whether ERK and JNK participate in hellebrigenin‐induced apoptosis, we employed an ERK inhibitor (U0126) and a JNK inhibitor (JNK‐in‐8) to measure the activity of cleaved caspases. A significant difference was noted in cleaved caspase‐3, caspase‐8 and PARP expression between cells treated with U0126 and hellebrigenin and cells treated with hellebrigenin alone (Figure 6C,D). Moreover, the expression levels of cleaved caspase‐3, caspase‐8 and PARP were upregulated in the group of combination of JNK‐in‐8 and hellebrigenin compared with hellebrigenin alone (Figure 6E,F). These findings suggested that hellebrigenin induced apoptosis in NPC cells through the inhibition of the ERK and JNK pathways.

### 3.6 Effect of Hellebrigenin on NPC Xenograft Mice

To assess the therapeutic potential of hellebrigenin in vivo, we established an NPC xenograft model in bulb/c nude mice. The mice were randomly divided into two groups that were treated with either vehicle control or 4 mg of hellebrigenin. As depicted in Figure 6G, hellebrigenin treatment significantly decreased tumour volume without affecting body weight (Figure 6H). Immunohistochemical analysis revealed the decreased expression of Ki67 and CHCHD2 in the hellebrigenin‐treated group, which reflected that hellebrigenin inhibited the tumourigenesis of NPC (Figure 6I). These findings suggest that hellebrigenin can effectively induce apoptosis by targeting CHCHD2 both in vitro and in vivo.

## 4 Discussion

This study investigated the effect of hellebrigenin on apoptosis in NPC cell lines, with a focus on its impact on CHCHD2 suppression. Our findings revealed that hellebrigenin significantly induced cell cycle arrest in the G2/M phase, induced cell cytotoxicity by initiating apoptosis in NPC cells and suppressed tumour progression in an NPC xenograft model. Hellebrigenin‐induced apoptosis was closely associated with the downregulation of MAPK pathways and CHCHD2 expression. A previous study indicated that hellebrigenin exhibits cytotoxic effects at the low doses of 2, 4 and 8 nM in the oral cancer cell lines SCC‐1 and SCC‐47 as well as in the human gingival epithelioid SG cell line [19]. Notably, the present study revealed that at higher concentrations (5, 10 and 20 nM), hellebrigenin significantly suppressed the viability of NPC‐039 and NPC‐BM cells without affecting normal nasopharyngeal cells, suggesting its selectivity for malignant cells. Consistent with previous findings in glioblastoma [9], hepatocellular carcinoma [20] and breast cancer [10], the present study demonstrated that 24‐h treatment with hellebrigenin induced cell cycle arrest in the G2/M phase in NPC cell lines. Moreover, a previous study reported that the downregulation of CDC2 and cyclin B1 expression may contribute to cell cycle arrest in the G2/M phase by inhibiting the activation of the CDC2–cyclin B1 complex [21]. Hellebrigenin has been demonstrated to be an effective apoptosis inducer in various tumour types. Our results indicate that hellebrigenin triggers apoptosis through extrinsic and intrinsic pathways, as evidenced by caspase activation and increased apoptotic cell populations in treated NPC cells.

CHCHD2, a member of the mitochondrial CHCH domain protein family, is primarily localised in the mitochondrial intermembrane space. Under stress conditions, it can translocate to the nucleus and function as a transcription factor for cytochrome c oxidase subunit 4 isoform 2 and CHCHD2 itself [22, 23]. Functionally, CHCHD2 regulates the mitochondrial membrane potential, reactive oxygen species and growth rate [22]. Liu et al. reported that together with Bcl‐xL, CHCHD2 inhibits the mitochondrial accumulation of Bax, reducing apoptosis [24]. Conversely, the lack of CHCHD2 in the mitochondria prevents Bcl‐xL and Bax binding, leading to Bax accumulation, oligomerisation, mitochondrial outer membrane permeabilisation and apoptosis [24]. This evidence aligns with our findings, which demonstrated the downregulation of both Bcl‐xL and CHCHD2 in hellebrigenin‐induced apoptotic NPC cells. CHCHD2 overexpression is correlated with various cancers. Yao et al. reported a significant association between CHCHD2 expression and lymph node metastasis, poor differentiation and poor prognosis in patients with hepatocellular carcinoma [25]. In patients with breast cancer, CHCHD2 was positively correlated with matrix metalloproteinase‐2, and high CHCHD2 expression was related to distant metastasis and poor prognosis [26].

Proteomic data revealed that, among the differentially expressed genes in two NPC cell lines treated with hellebrigenin, 17 genes overlapped with those showing higher expression levels in NPC patients within the GSE53819 data set. Based on insights from GO and KEGG pathway analyses, we hypothesized that the genes regulated by hellebrigenin may be associated with mitochondrial function. Consequently, we further investigated the role of CHCHD2 in head and neck cancers as well as NPC. The GEO database demonstrated the higher expression level of CHCHD2 in NPC patients compared with normal nasopharynx tissue. The TCGA database revealed a correlation between CHCHD2 expression and advanced stage and poor prognosis in patients with HNSCC. Moreover, in the present study, the use of the CHCHD2 plasmid and siRNA in double‐stained apoptotic assays and Western blot assays confirmed the direct involvement of CHCHD2 in apoptosis in NPC‐039 and NPC‐BM cell lines. Collectively, these findings highlight the crucial role of CHCHD2 in hellebrigenin‐induced apoptosis in NPC cells.

MAPK cascades, including MAPK kinase kinase, MAPK kinase and MAPK, phosphorylate downstream proteins to activate signal transduction [27]. MAPKs are frequently dysregulated in cancer cells, and they play crucial roles in cellular processes. ERK1/2 primarily regulates cell survival and proliferation, whereas p38 and JNK are involved in apoptosis regulation [28]. In the present study, the examination of the effects of cotreatment with hellebrigenin and ERK or JNK inhibitors indicated that hellebrigenin induced apoptosis through the suppression of JNK and ERK pathways. These findings align with previous findings demonstrating that hellebrigenin can inhibit JNK, ERK and p38 pathways and can induce apoptosis in breast cancer cells [10]. Liu et al. reported the in vivo antitumor effects of hellebrigenin [29], and they also indicated that at a dose of 2.5 mg/kg, hellebrigenin reduced tumour weight and induced alterations in the expression of apoptotic proteins, including the upregulation of cleaved caspase‐3, caspase‐9 and Bax and the downregulation of Bcl‐2. In our study, hellebrigenin significantly reduced the tumour volume in the NPC xenograft model, and Ki67 and CHCHD2 expression were reduced in hellebrigenin‐treated tumours. These data indicate that hellebrigenin induced apoptosis through CHCHD2 suppression both in vitro and in vivo.

There are limitations in the present study. Due to adherence to the ‘3R principles’, specifically the reduction of animal use, only three mice were included in each group as samples. This small sample size may limit the statistical power of the study. Additionally, the experimental design lacked a high‐dose group, which should be included in future experiments to investigate dose‐dependent effects and toxicity thresholds. Moreover, the clinical application of hellebrigenin is limited due to its Na^+^/K^+^‐ATPase binding activity [30]. To address the cardiotoxicity of bufadienolides, targeted drug delivery systems have been developed to enhance their bioavailability in several studies. For instance, bafalin‐loaded MnO_2_ hollow nanoparticles coated with platelet membranes demonstrated 3.5‐fold higher efficiency for tumour growth inhibition than free bafalin [31]. Additionally, bafalin‐loaded pluronic polyetherimide nanoparticles significantly reduced tumour volume in a colon cancer xenograft model [32]. Although research on hellebrigenin‐specific drug delivery systems is limited, existing studies on bufadienolides in comparable delivery platforms [33] may provide valuable guidance for future investigations into the targeted application of hellebrigenin.

Nevertheless, while the current study adopts a conventional molecular and cellular approach—reflective of the prevailing somatic mutation‐centric paradigm—it is increasingly recognised that this perspective alone may be insufficient to address complex clinical issues such as recurrence and drug resistance [34, 35]. NPC is now understood not merely as a genetic disease, but as a dynamic pathological ecosystem shaped by ecological and evolutionary processes [36]. In particular, the tumour microenvironment (TME)—comprising stromal cells, immune components and abiotic factors—plays a crucial role in disease progression, metastasis and therapeutic resistance. Our present investigation focuses primarily on intrinsic cellular mechanisms and does not encompass these broader ecological interactions. Future studies are warranted to explore how hellebrigenin modulates the tumour microenvironment and the ecological relationships within it. Such efforts may inform the development of novel therapeutic strategies grounded in an ecological understanding of cancer biology.

## 5 Conclusion

This study provides novel evidence demonstrating that hellebrigenin induces apoptosis in NPC cells through CHCHD2 suppression both in vitro and in vivo. Additionally, this study reveals that the downregulation of ERK and JNK signalling pathways mediates hellebrigenin‐induced apoptosis. This represents the first demonstrated association between hellebrigenin‐induced apoptosis and CHCHD2. Thus, hellebrigenin can be developed as a potential therapeutic agent for NPC. Further research is warranted to explore the potential benefits of combining hellebrigenin with existing chemotherapeutic agents for reducing side effects and enhancing therapeutic efficacy.

## Author Contributions

**Hsin‐Yu Ho:** methodology (equal), software (equal), writing – original draft (equal), writing – review and editing (equal). **Mu‐Kuan Chen:** conceptualization (equal), writing – review and editing (equal). **Yun‐Jung Tsai:** methodology (equal), software (equal). **Chia‐Chieh Lin:** methodology (equal), software (equal). **Yu‐Sheng Lo:** methodology (equal), software (equal). **Yi‐Ching Chuang:** methodology (equal), software (equal). **Ming‐Ju Hsieh:** conceptualization (equal), writing – original draft (equal), writing – review and editing (equal).

## Conflicts of Interest

The authors declare no conflicts of interest.

## Data Availability

The data used to support the findings of this study are available from the corresponding author upon request.
